# Antidiabetic medications and risk of cognitive disorders in type 2 diabetes: A retrospective cohort study

**DOI:** 10.1371/journal.pone.0356138

**Published:** 2026-08-24

**Authors:** Ahmad Basil Nasir, Yassine Kilani, Mohammad Aldiabat, Omar Abdelghany, Youssef Hafez, Nitin Desai, Mahmoud Y. Madi, Francis G. Wade, Kamran Qureshi, Lewis J. Frey, Adam D. Farmer, Wing-Kin Syn

**Affiliations:** 1 Division of Gastroenterology and Hepatology, Department of Internal Medicine, Saint Louis University School of Medicine, Saint Louis, Missouri, United States of America; 2 Department of Internal Medicine, Saint Louis University School of Medicine, Saint Louis, Missouri, United States of America; 3 Department of Internal Medicine, Washington University, Saint Louis, Missouri, United States of America; 4 Department of Pathology, Saint Louis University School of Medicine, Saint Louis, Missouri, United States of America; 5 VA St. Louis Healthcare System, St. Louis, Missouri, United States of America; 6 Department of Physiology, Faculty of Medicine and Nursing, University of the Basque Country (UPV/EHU), Vizcaya, Spain; Medical School, University of Zagreb, CROATIA

## Abstract

**Objective:**

To evaluate the associations between commonly used antidiabetic regimens and incidence of mild cognitive disorder, Alzheimer disease, and vascular dementia in adults with type 2 diabetes.

**Design, setting, and participants:**

This retrospective cohort study used the TriNetX US Collaborative Network of electronic health records from 2010 to 2024. Adults aged 40–69 years with type 2 diabetes and at least 1 year of follow-up were included. Propensity score matching was applied to balance covariates.

**Exposure:**

Patients were classified into 5 treatment groups: metformin only (reference), metformin plus DPP-4 inhibitors, metformin plus GLP-1 receptor agonists, metformin plus SGLT-2 inhibitors, and insulin monotherapy. Exposure was defined by first recorded prescription and continued use during follow-up.

**Main outcomes and measures:**

Primary outcomes were incident mild cognitive disorder, Alzheimer disease, and vascular dementia, identified using ICD-10 codes. Hazard ratios with 95% confidence intervals were estimated from Cox proportional hazards models, with landmark analyses for follow-up shorter and longer than 5 years.

**Results:**

Among 1,528,885 adults with type 2 diabetes (mean age, 58 years; 49.2% women), GLP-1 receptor agonists plus metformin were associated with lower incidence of vascular dementia (HR, 0.46; 95% CI, 0.37–0.57), mild cognitive disorder (HR, 0.79; 95% CI, 0.66–0.95), and Alzheimer disease (HR, 0.46; 95% CI, 0.31–0.70). SGLT-2 inhibitors plus metformin reduced vascular dementia risk (HR, 0.68; 95% CI, 0.54–0.87) but not other outcomes. Insulin monotherapy was associated with higher incidence of vascular dementia (HR, 3.27; 95% CI, 3.03–3.52), mild cognitive disorder (HR, 1.71; 95% CI, 1.56–1.87), and Alzheimer disease (HR, 1.56; 95% CI, 1.32–1.84).

**Conclusions and Relevance:**

GLP-1 receptor agonists and SGLT-2 inhibitors combined with metformin were associated with reduced risk of cognitive decline. Insulin monotherapy was associated with higher incidence of all three outcomes; however, this association is likely influenced by confounding by indication, unmeasured markers of diabetes severity (including diabetes duration, cardiovascular and renal disease severity, and microvascular and macrovascular complications), and shorter follow-up among insulin users, and should not be interpreted as a direct drug effect. Because standard Cox models do not account for death as a competing event, reported hazard ratios reflect cause-specific hazards and may not directly correspond to cumulative incidence, particularly for the insulin group, in which mortality and censoring were substantially higher. Antidiabetic medication choice may influence long-term cognitive outcomes and should be considered in diabetes management.

## Introduction

Diabetes is a chronic progressive condition projected to affect more than 1.3 billion people worldwide by 2050 [1,2]. Type 2 diabetes mellitus (T2DM) nearly doubles the risk of cognitive decline, including mild cognitive disorder (MCD), Alzheimer disease (AD), and vascular dementia (VD) [3]. Several mechanisms have been implicated, including hyperglycemia, insulin resistance, microvascular injury, and neuroinflammation [4–6]. Both hyperglycemia and hypoglycemia are associated with impaired cognition [7,8], emphasizing the importance of treatment selection.

Observational studies suggest some antidiabetic agents may confer neuroprotective effects. Metformin has been linked to slower cognitive decline and reduced dementia risk [9–11]. However, some studies have reported conflicting findings, with long-term metformin use associated with increased Alzheimer disease risk, possibly mediated through vitamin B12 depletion and subsequent hyperhomocysteinemia [12–15]. GLP-1 receptor agonists enhance brain insulin signaling, reduce amyloid accumulation, and mitigate neuroinflammation [16–18]. SGLT-2 inhibitors may improve vascular outcomes relevant to dementia risk, [19] while findings for DPP-4 inhibitors have been inconsistent [20, 21]. In contrast, insulin therapy, often associated with hypoglycemia and advanced disease, has been linked to greater risk of cognitive impairment [22–24].

However, most prior studies were limited by small sample sizes, short follow-up, or a focus on a single drug class, restricting generalizability [25,26]. Large-scale analyses directly comparing multiple antidiabetic medications with long-term cognitive outcomes remain scarce.

The aim of this study was to evaluate the association between commonly used antidiabetic regimens — metformin monotherapy, metformin plus DPP-4 inhibitors, metformin plus GLP-1 receptor agonists, metformin plus SGLT-2 inhibitors, and insulin monotherapy — and the incidence of mild cognitive disorder, Alzheimer disease, and vascular dementia in a large, real-world cohort of adults with type 2 diabetes.

## Methods

### Study design and data source

This retrospective cohort study was conducted and reported in accordance with the Strengthening the Reporting of Observational Studies in Epidemiology (STROBE) guidelines. We conducted a retrospective cohort study using the TriNetX US Collaborative Network, which contains deidentified electronic health records from more than 118 million patients across 69 health care organizations. The database includes demographic, diagnostic, laboratory, and prescription data coded using the International Classification of Diseases, Ninth and Tenth Revisions (ICD-9 and ICD-10). Analyses were conducted within the TriNetX platform, which provides built-in analytic tools for cohort creation and outcome comparisons.

### Participants

Adults aged 40–69 years with a diagnosis of type 2 diabetes mellitus were eligible if they had at least 1 year of follow-up after the index date, defined as the first prescription of an antidiabetic medication. Patients who subsequently added a second agent were classified based on their first recorded combination regimen. Patients with short-term metformin use followed by transition to combination therapy were classified in the combination group at the time of the combination prescription. Exclusion criteria included patients with Type 1 diabetes mellitus (UMLS: ICD10 CM:E10); or Human immunodeficiency virus disease (UMLS: ICD10 CM:B20); or pre-existing diagnoses of dementia or cognitive impairment and incomplete demographic or clinical data.

### Exposures

Patients were classified into 5 mutually exclusive treatment groups based on their first recorded antidiabetic regimen: metformin only (reference), metformin plus dipeptidyl peptidase-4 (DPP-4) inhibitors, metformin plus glucagon-like peptide-1 (GLP-1) receptor agonists, metformin plus sodium-glucose cotransporter-2 (SGLT-2) inhibitors, and insulin monotherapy. Exposure was defined by initiation of the index regimen with continued use during the follow-up period.

### Outcomes

The primary outcomes were incident diagnoses of mild cognitive disorder, Alzheimer disease, and vascular dementia, identified using ICD-10 codes (G31.84, G30, and F01, respectively). Outcomes were assessed beginning 1 day after the index date until loss to follow-up or the end of the study period. Analyses were stratified by follow-up duration of less than 5 years and 5 years or longer.

### Statistical analyses

Baseline characteristics were summarized as means with standard deviations for continuous variables and proportions for categorical variables. Propensity score matching (1:1) was performed using a greedy nearest-neighbor algorithm with a caliper width of 0.1 pooled standard deviations. Covariates included age, sex, race, ethnicity, sleep apnea, hypothyroidism, tobacco use, alcohol use, head injury, glucocorticoid use, and glycated hemoglobin. Cardiovascular disease and renal disease were not included as propensity score covariates due to the complexity of capturing these heterogeneously coded conditions within the TriNetX platform; their potential confounding influence is acknowledged as a study limitation. Balance was assessed using standardized mean differences (<0.1 indicating adequate balance). Hazard ratios with 95% confidence intervals were estimated using Cox proportional hazards models. Proportionality was evaluated with scaled Schoenfeld residuals, and landmark analyses were conducted at less than 5 years and 5 years or longer. The Benjamini-Hochberg method was applied to control the false discovery rate. Two-sided

*P* < 0.05 was considered statistically significant. Because standard Cox proportional hazards models estimate cause-specific hazard ratios rather than the cumulative incidence of the outcome, the hazard ratios reported here reflect the instantaneous risk of the diagnosed outcome among patients who remain under observation (i.e., have not died or otherwise been censored), rather than the absolute probability of developing the outcome over the follow-up period. This distinction is particularly relevant for the insulin monotherapy group, in which competing mortality risk is expected to be substantially higher. Mean and median follow-up duration were calculated for each treatment group (Table 1).

**Table 1:** Mean and Median Follow-up Duration After Propensity Score Matching by Treatment Group and Landmark Period.

Treatment Comparison	Group	After Matching <5 Years		After Matching ≥5 Years	
		Mean (days)	Median (days)	Mean (days)	Median (days)
**Metformin + DPP-4i vs Metformin Only**	Metformin + DPP-4i	838	694	1,038	694
	Metformin Only	839	697	1,057	697
**Metformin + GLP-1 RA vs Metformin Only**	Metformin + GLP-1 RA	618	489	665	489
	Metformin Only	526	363	567	363
**Metformin + SGLT-2i vs Metformin Only**	Metformin + SGLT-2i	571	406	601	406
	Metformin Only	533	356	565	356
**Insulin Monotherapy vs Metformin Only**	Insulin Monotherapy	608	303^ᵃ^	754	303^ᵃ^
	Metformin Only	791	612	980	612

All values reflect follow-up duration after 1:1 propensity score matching. Follow-up was censored at the last recorded fact in each patient’s electronic health record. The landmark period of less than 5 years includes outcomes occurring 1–1,825 days after the index date; the landmark period of 5 years or longer includes outcomes occurring after 1,825 days.

ᵃInsulin monotherapy users had a 2-fold shorter median follow-up than matched metformin-only users across both landmark periods (303 days vs 612 days), consistent with higher rates of censoring attributable to more advanced disease severity and competing mortality risk.DPP-4i = dipeptidyl peptidase-4 inhibitors, GLP-1 RA = glucagon-like peptide-1 receptor agonists, SGLT-2i = sodium-glucose cotransporter-2 inhibitors.

### Patient and public involvement

Patients or the public were not involved in the design, conduct, reporting, or dissemination plans of this research.

### Ethical approval

The TriNetX platform provides access to deidentified patient data in compliance with the Health Insurance Portability and Accountability Act. Because only aggregate, deidentified data were used, the Saint Louis University Institutional Review Board determined that this study did not constitute human subjects research and was exempt from review.

## Results

**Fig 1:**
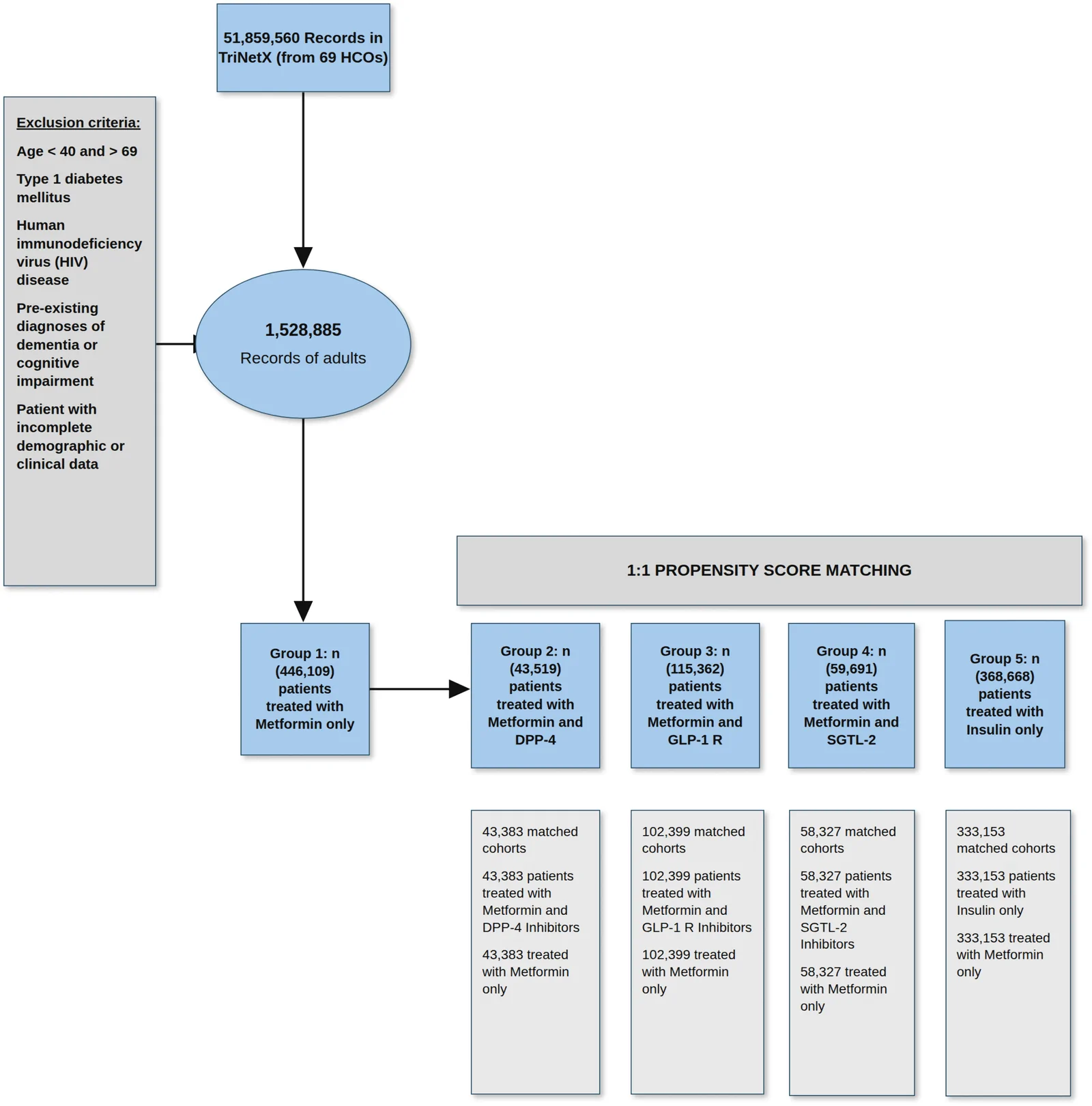
Flowchart showing the procedures followed in creating the unmatched original cohorts and the 1:1 matched groups from TriNetX Network Platform.

Of 51,859,560 patient records screened, 1,528,885 adults aged 40–69 years with type 2 diabetes were eligible (mean age, 58 years; 49.2% women). Treatment groups included 446,109 receiving metformin only, 43,519 receiving metformin plus DPP-4 inhibitors, 115,362 receiving metformin plus GLP-1 receptor agonists, 59,691 receiving metformin plus SGLT-2 inhibitors, and 368,668 receiving insulin monotherapy. After 1:1 propensity score matching, comparator cohorts included 43,383 patients for DPP-4 inhibitors (0.3% of DPP-4 inhibitor patients unmatched), 102,399 for GLP-1 receptor agonists (11.2% unmatched), 58,327 for SGLT-2 inhibitors (2.3% unmatched), and 333,153 for insulin (9.6% unmatched), each matched to equal-sized metformin-only groups (Fig 1).

Before matching, treatment groups differed in demographic and clinical features. Patients receiving DPP-4 inhibitors were older, those receiving GLP-1 receptor agonists were more frequently female with higher rates of sleep apnea and glucocorticoid use, and those receiving SGLT-2 inhibitors were more frequently male. Insulin users had higher prevalence of alcohol abuse. After matching, baseline characteristics were well balanced, with standardized mean differences <0.1 for most variables. Minor residual imbalances in hemoglobin A1c persisted after matching, with standardized mean differences of 0.164 for the DPP-4 inhibitor group, 0.152 for the SGLT-2 inhibitor group, 0.079 for the insulin group, and 0.021 for the GLP-1 receptor agonist group. The hemoglobin A1c imbalance in the DPP-4 inhibitor and SGLT-2 inhibitor comparisons exceeded the conventional threshold of 0.1, indicating residual glycemic confounding that may influence results for these groups and should be considered when interpreting the findings. Baseline characteristics are summarized in Supplementary (S1–S4 Tables). Mean and median follow-up duration after propensity score matching for all treatment groups and landmark periods are summarized in (Table 1). Notably, insulin monotherapy users had substantially shorter median follow-up than matched metformin-only users across both landmark periods (303 days vs 612 days), consistent with higher rates of censoring attributable to more advanced disease severity and competing mortality risk.

**Fig 2:**
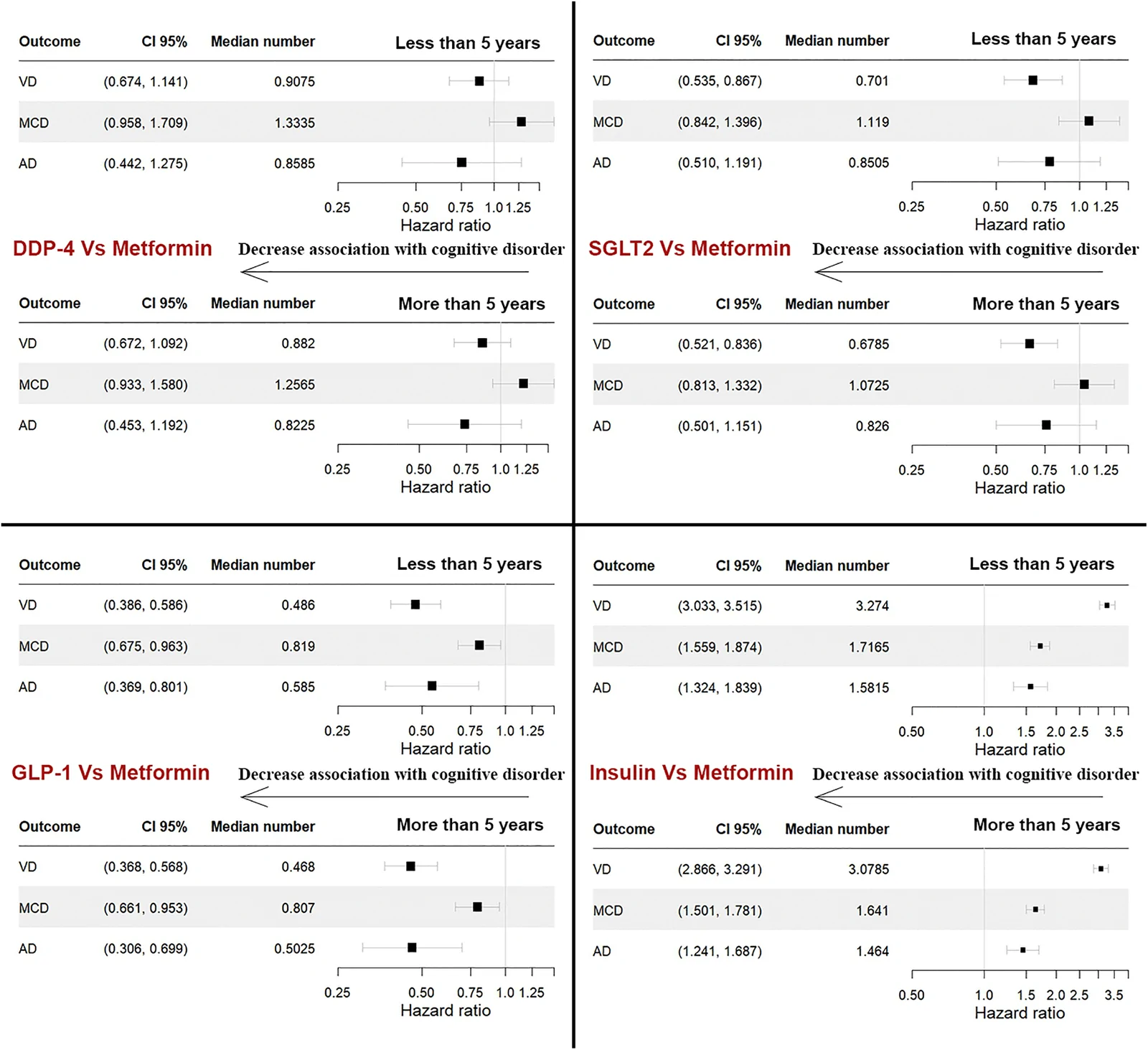
Propensity score–matched hazard ratios for vascular dementia, mild cognitive disorder, and Alzheimer disease comparing metformin plus DPP-4 inhibitors, metformin plus GLP-1 receptor agonists, metformin plus SGLT-2 inhibitors, and insulin monotherapy vs metformin alone, stratified by follow-up duration (<5 years vs ≥ 5 years).

In matched analyses, GLP-1 receptor agonists plus metformin (n = 102,399) were associated with lower incidence of vascular dementia (124 vs 243 cases; HR, 0.46; 95% CI, 0.37–0.57), mild cognitive disorder (216 vs 242; HR, 0.79; 95% CI, 0.66–0.95), and Alzheimer disease (34 vs 66; HR, 0.46; 95% CI, 0.31–0.70) compared with metformin alone (n = 102,399). These associations were consistent at <5 years and ≥5 years of follow-up (Table 2; Fig 2).

**Table 2:** Cognitive outcomes in patients receiving metformin plus DPP-4 inhibitors or metformin plus GLP-1 receptor agonists compared with metformin only, after propensity score matching.

		Metformin + DPP-4i (N = 43,383 matched pairs)					Metformin + GLP-1 RA (N = 102,399 matched pairs)				
Period	Outcome	Cases (DPP-4i + Metf)	Cases (Metformin)	HR	95% CI	p	Cases (Metf + GLP-1 RA)	Cases (Metformin)	HR	95% CI	p
<5 years	Vascular dementia	104	119	0.877	(0.674-1.141)	0.329	124	243	0.457	(0.368–0.568)	<0.001
	Mild cognitive disorder	104	82	1.279	(0.958-1.709)	0.095	216	242	0.794	(0.661–0.953)	0.013
	Alzheimer disease	24	32	0.751	(0.442-1.275)	0.287	34	66	0.462	(0.306–0.699)	<0.001
≥5 years	Vascular dementia	119	144	0.856	(0.672-1.092)	0.211	135	255	0.475	(0.386–0.586)	<0.001
	Mild cognitive disorder	120	103	1.214	(0.933-1.580)	0.149	231	256	0.806	(0.675–0.963)	0.017
	Alzheimer disease	28	40	0.735	(0.453-1.192)	0.210	41	68	0.544	(0.369–0.801)	0.002

Each combination group was matched 1:1 to a metformin-only comparator. Landmark analysis at <5 years and ≥5 years of follow-up. Unadjusted pre-matching outcome comparisons are available in Supplementary S5 Table. HR = hazard ratio; CI = confidence interval; DPP-4i = dipeptidyl peptidase-4 inhibitors; GLP-1 RA = glucagon-like peptide-1 receptor agonists; Metf. = metformin. Cox proportional hazards models used for all estimates.

The absolute risk difference for vascular dementia with metformin plus GLP-1 receptor agonists was −0.93 per 1,000 person-years at less than 5 years and −0.88 per 1,000 person-years at 5 years or longer, indicating that approximately 1 fewer case of vascular dementia occurs per 1,000 patients treated per year compared with metformin alone (Table 4).

SGLT-2 inhibitors plus metformin (n = 58,327) were associated with lower incidence of vascular dementia at <5 years (114 vs 157 cases; HR, 0.68; 95% CI, 0.54–0.87) and ≥5 years (117 vs 168; HR, 0.66; 95% CI, 0.52–0.84), but were not significantly associated with mild cognitive disorder or Alzheimer disease (Table 3; Fig 2).

**Table 3:** Cognitive outcomes in patients receiving metformin plus SGLT-2 inhibitors or insulin monotherapy compared with metformin only, after propensity score matching.

		Metformin + SGLT-2i (N = 58,327 matched pairs)					Insulin monotherapy (N = 333,153 matched pairs)				
Period	Outcome	Cases (Metf + SGLT-2i)	Cases (Metformin)	HR	95% CI	p	Cases (Insulin)	Cases (Metformin)	HR	95% CI	p
<5 years	Vascular dementia	114	157	0.681	(0.535–0.867)	0.002	2,630	966	3.265	(3.033–3.515)	<0.001
	Mild cognitive disorder	130	112	1.084	(0.842–1.396)	0.531	1,088	781	1.709	(1.559–1.874)	<0.001
	Alzheimer disease	39	47	0.779	(0.510–1.191)	0.248	327	254	1.561	(1.324–1.839)	<0.001
≥5 years	Vascular dementia	117	168	0.660	(0.521–0.836)	0.001	2,848	1,122	3.071	(2.866–3.291)	<0.001
	Mild cognitive disorder	133	120	1.041	(0.813–1.332)	0.751	1,222	925	1.635	(1.501–1.781)	<0.001
	Alzheimer disease	40	50	0.759	(0.501–1.151)	0.193	356	301	1.447	(1.241–1.687)	<0.001

Each treatment group was matched 1:1 to a metformin-only comparator. Insulin monotherapy was used without metformin. Landmark analysis at <5 years and ≥5 years of follow-up. Unadjusted pre-matching outcome comparisons are available in Supplementary S6 Table. HR = hazard ratio; CI = confidence interval; SGLT-2i = sodium-glucose cotransporter-2 inhibitors; Metf. = metformin only. Cox proportional hazards models used for all estimates.

DPP-4 inhibitors plus metformin (n = 43,383) were not significantly associated with vascular dementia, mild cognitive disorder, or Alzheimer disease at either follow-up interval (Table 2; Fig 2).

In contrast, insulin monotherapy (n = 333,153) was associated with higher incidence of all outcomes compared with matched metformin-only patients. Risks were increased for vascular dementia (2,630 vs 966 cases; HR, 3.27; 95% CI, 3.03–3.52 at <5 years; 2,848 vs 1,122 cases; HR, 3.07; 95% CI, 2.87–3.29 at ≥5 years), mild cognitive disorder (1,088 vs 781 cases; HR, 1.71; 95% CI, 1.56–1.87 at <5 years; 1,222 vs 925 cases; HR, 1.64; 95% CI, 1.50–1.78 at ≥5 years), and Alzheimer disease (327 vs 254 cases; HR, 1.56; 95% CI, 1.32–1.84 at <5 years; 356 vs 301 cases; HR, 1.45; 95% CI, 1.24–1.69 at ≥5 years) (Table 3; Fig 2).

The absolute risk difference for vascular dementia with insulin monotherapy was + 3.40 per 1,000 person-years at less than 5 years and +2.88 per 1,000 person-years at 5 years or longer, indicating that approximately 3 additional cases of vascular dementia occur per 1,000 insulin-treated patients per year compared with metformin alone (Table 4).

**Table 4:** Absolute Risk Differences per 1,000 Person-Years After Propensity Score Matching.

Outcome	Time Window	ARD per 1,000 Person-Years			
		Metformin + DPP-4i	Metformin + GLP-1 RA	Metformin + SGLT-2i	Insulin Monotherapy
**Vascular Dementia**	<5 years	−0.15	−0.93	−0.59	+3.40
	≥5 years	−0.18	−0.88	−0.64	+2.88
**Mild Cognitive Disorder**	<5 years	+0.22	−0.39	+0.11	+0.88
	≥5 years	+0.15	−0.37	+0.06	+0.75
**Alzheimer Disease**	<5 years	−0.08	−0.25	−0.12	+0.24
	≥5 years	−0.09	−0.21	−0.13	+0.18

ARD = Absolute Risk Difference, calculated as the incidence rate of the comparator group minus the incidence rate of the metformin-only group, per 1,000 person-years. Negative values indicate lower incidence in the comparator group; positive values indicate higher incidence ARD was calculated using mean follow-up duration from Table 1. DPP-4i = dipeptidyl peptidase-4 inhibitors, GLP-1 RA = glucagon-like peptide-1 receptor agonists, MCD = mild cognitive disorder, SGLT-2i = sodium-glucose cotransporter-2 inhibitors.

### Sensitivity analyses

Findings were consistent across short-term (<5 years) and long-term (≥5 years) follow-up. GLP-1 receptor agonists plus metformin were associated with reduced risk of all outcomes, SGLT-2 inhibitors plus metformin were consistently associated with reduced risk of vascular dementia only, DPP-4 inhibitors were not significantly associated with cognitive outcomes, and insulin monotherapy was consistently associated with increased risk for all outcomes (Table 2,3; Fig 1,2).

## Discussion

In this cohort of more than 1.5 million adults with type 2 diabetes, use of GLP-1 receptor agonists plus metformin was associated with lower incidence of vascular dementia, mild cognitive disorder, and Alzheimer disease. SGLT-2 inhibitors plus metformin were associated with reduced risk of vascular dementia only, and DPP-4 inhibitors showed no significant associations. Insulin monotherapy was associated with substantially higher risk of all cognitive outcomes, with effects consistent across short- and long-term follow-up.

These findings reinforce growing evidence that GLP-1 receptor agonists may provide neuroprotection. Mechanistic studies demonstrate improvements in brain insulin signaling, reductions in neuroinflammation, and enhanced amyloid clearance [16–18]. Observational data and meta-analyses report reduced dementia risk among GLP-1 users, with pooled estimates ranging from OR 0.58 to RR 0.72 [17,18,27]. Our hazard ratios (0.46–0.79) are consistent with these results and extend prior work by demonstrating benefit across multiple cognitive outcomes. The combined use of GLP-1 receptor agonists with metformin—an agent itself linked to reduced dementia risk [28–30], may partly explain the stronger associations observed here.

The neuroprotective mechanisms of GLP-1 receptor agonists are multifaceted. These agents cross the blood-brain barrier and act on GLP-1 receptors expressed in the hippocampus, cortex, and hypothalamus [20,21,26]. Preclinical studies demonstrate reductions in amyloid-beta accumulation and tau phosphorylation, attenuation of neuroinflammation through suppression of microglial activation, enhancement of synaptic plasticity, and promotion of neurogenesis [26,31]. Additionally, GLP-1 receptor agonists improve cerebral blood flow and reduce oxidative stress, both of which are relevant to vascular dementia pathophysiology [17,18,21].

SGLT-2 inhibitors have been proposed to benefit cerebrovascular health through improvements in glycemia, blood pressure, and endothelial function, and reduce glucotoxicity and oxidative stress — all of which may contribute to cerebrovascular protection. Animal models have demonstrated reductions in neuroinflammatory markers and improvements in mitochondrial function with SGLT-2 inhibitor treatment [19]. Prior studies have yielded mixed findings [31,32]. In this study, SGLT-2 inhibitors were consistently associated with reduced risk of vascular dementia, but not with Alzheimer disease or mild cognitive disorder.

Evidence for DPP-4 inhibitors has been inconsistent, with some reports suggesting neutral effects and others modest benefit. DPP-4 inhibitors increase endogenous GLP-1 and GIP levels and have demonstrated anti-inflammatory and neuroprotective properties in vitro and in animal models. However, the magnitude of GLP-1 elevation achieved with DPP-4 inhibitors is substantially lower than that achieved with GLP-1 receptor agonists, which may explain the lack of meaningful cognitive benefit observed in clinical studies including the present one [20,21,33]. Our findings support the likelihood of no clinically meaningful association.

Insulin therapy has been linked to dementia risk, possibly through recurrent hypoglycemia, more advanced disease, and direct neurobiological effects [22–24,34,35]. Large cohorts have shown increased incidence of both vascular and Alzheimer-type dementia among insulin users [34,35]. Our results are consistent, demonstrating more than a threefold higher risk of vascular dementia and significantly increased risk of mild cognitive disorder and Alzheimer disease with insulin monotherapy.

The markedly elevated risk observed with insulin monotherapy should be interpreted with caution. Insulin is preferentially prescribed in patients with longer disease duration, poorer glycemic control, greater comorbidity burden, and higher rates of microvascular and macrovascular complications. Despite propensity score matching, these differences in disease severity are difficult to balance using administrative data alone. Residual confounding by indication therefore remains the most likely explanation for the magnitude of the observed associations, and these findings should not be interpreted as evidence of a direct neurotoxic effect of insulin [36,37].

The relationship between hypoglycemia and cognitive impairment appears to be bidirectional — hypoglycemia may contribute to dementia risk, but cognitive impairment also predisposes patients to hypoglycemic events through impaired diabetes self-management. This bidirectionality complicates causal inference and suggests that hypoglycemia in insulin-treated patients may, in part, represent a consequence rather than a cause of early cognitive dysfunction [38–40].

An important analytical consideration is the role of competing mortality risk. Patients receiving insulin therapy, who tend to have more advanced disease, face elevated cardiovascular and all-cause mortality risk. Death before dementia onset represents a competing event that standard Cox proportional hazards models do not account for; the direction and magnitude of this effect on the observed associations is not necessarily uniform and could complicate interpretation of the relative hazard ratios, particularly for insulin. Consistent with this concern, the median follow-up in the insulin group was 303 days compared with 612 days in the matched metformin group — a two-fold difference — suggesting that insulin users were censored earlier, likely due to death or loss to follow-up related to more advanced disease. Formal competing risk analyses were not performed in this study and represent an important methodological limitation; the reported hazard ratios should therefore be interpreted as cause-specific hazards rather than estimates of cumulative incidence.

Strengths of this study include the large, diverse sample; evaluation of multiple antidiabetic drug classes; use of propensity score matching; and availability of long-term follow-up.

Several limitations warrant consideration. The observational design cannot establish causality, and residual confounding is possible despite rigorous matching. Diagnoses were identified by ICD-10 codes, which may be subject to misclassification, and cognitive outcomes may be underdiagnosed in clinical settings, leading to underestimation of true incidence. Medication adherence and duration of exposure could not be verified. Although TriNetX captures data from diverse US health systems, results may not generalize to populations outside the US. Confounding by indication is a central concern particularly for the insulin group, as insulin is typically prescribed in patients with more advanced, longer-duration diabetes and greater comorbidity burden — factors that independently increase dementia risk. Propensity score matching did not include cardiovascular disease severity, renal function, diabetes duration, APOE ε4 genotype, education level, physical activity, or socioeconomic status, all of which are relevant to dementia risk. Furthermore, residual hemoglobin A1c imbalance after matching (SMD 0.164 for DPP-4 inhibitors and 0.152 for SGLT-2 inhibitors, both exceeding the 0.1 threshold) indicates that glycemic confounding may be present in these comparisons specifically. Mild cognitive disorder is known to be inconsistently coded in administrative databases, and ICD-10-based diagnoses may underdetect early or mild disease with variability across institutions. The absence of a competing risk framework means that differential mortality across groups — particularly for insulin users — may have influenced outcome ascertainment. The study population was restricted to adults aged 40–69 years, limiting generalizability to older adults who represent the highest-risk group for dementia. Finally, since all combination therapy groups included metformin as a backbone agent, any potential negative cognitive effect of metformin — such as through vitamin B12 depletion and hyperhomocysteinemia — could attenuate the apparent benefits of add-on agents; future studies using metformin-free comparator arms would help isolate the independent effects of each drug class [12–15,28,29].

## Conclusion

This study highlights the differential cognitive outcomes associated with various antidiabetic medications in patients with type 2 diabetes. GLP-1 receptor agonists combined with metformin were associated with reduced risk across all three outcomes — mild cognitive disorder, Alzheimer disease, and vascular dementia. SGLT-2 inhibitors combined with metformin showed a selective benefit for vascular dementia only, with no significant association with Alzheimer disease or mild cognitive disorder. DPP-4 inhibitors were not significantly associated with any cognitive outcome. Insulin monotherapy was associated with substantially increased risk of all three outcomes; however, this comparison is particularly difficult to interpret given the absence of key severity markers (diabetes duration, cardiovascular and renal disease severity, and micro- and macrovascular complications) and the substantially shorter follow-up among insulin users relative to matched metformin-only comparators. This finding likely reflects residual confounding by indication rather than a direct drug effect and should be interpreted with corresponding caution. These findings have potential clinical relevance for antidiabetic drug selection in adults with type 2 diabetes who are at risk for cognitive decline. When glycemic control goals can be achieved with multiple therapeutic options, the differential cognitive risk profiles of these agents — particularly the potential benefit of GLP-1 receptor agonists and SGLT-2 inhibitors combined with metformin — may inform individualized treatment decisions. However, prospective randomized trials are essential before practice-changing recommendations can be made.

## Supporting information

S1 Table Baseline characteristics: Metformin and DPP-4 inhibitors (Group 2) vs Metformin only (Group 1). (DOCX)

S2 Table Baseline characteristics: Metformin and GLP-1 receptor agonists (Group 3) vs Metformin only (Group 1). (DOCX)

S3 Table Baseline characteristics: Metformin and SGLT-2 inhibitors (Group 4) vs Metformin only (Group 1). (DOCX)

S4 Table Baseline characteristics: Insulin only (Group 5) vs Metformin only (Group 1). (DOCX)

S5 Table Cognitive outcomes in patients receiving metformin plus DPP-4 inhibitors compared with metformin only, before propensity score matching (unmatched cohorts; N = 446,109 comparator group). (DOCX)

S6 Table Cognitive outcomes in patients receiving metformin plus SGLT-2 inhibitors compared with metformin only, before propensity score matching (unmatched cohorts; N = 446,109 comparator group). (DOCX)

## References

[1] NCD Risk Factor Collaboration (NCD-RisC). Worldwide trends in diabetes prevalence and treatment from 1990 to 2022: a pooled analysis of 1108 population-representative studies with 141 million participants. Lancet. 2024;404(10467):2077–93. doi: 10.1016/S0140-6736(24)02317-1 39549716 PMC7616842

[2] Magliano DJ, Boyko EJ. IDF Diabetes Atlas. 10 ed. IDF Diabetes Atlas Committee. Brussels, Belgium: International Diabetes Federation. 2021.

[3] Jash K, Gondaliya P, Kirave P, Kulkarni B, Sunkaria A, Kalia K. Cognitive dysfunction: A growing link between diabetes and Alzheimer’s disease. Drug Dev Res. 2020;81(2):144–64. doi: 10.1002/ddr.21579 31820484

[4] Chauhan A, Dubey S, Jain S. Association Between Type 2 Diabetes Mellitus and Alzheimer’s Disease: Common Molecular Mechanism and Therapeutic Targets. Cell Biochem Funct. 2024;42(7):e4111. doi: 10.1002/cbf.4111 39228117

[5] Ciudin A, Hernández C. Diabetes-related cognitive impairment and dementia. Chronic Complications of Diabetes Mellitus. Elsevier. 2024. p. 215–30. doi: 10.1016/b978-0-323-88426-6.00020-8

[6] Duarte JMN. Loss of brain energy metabolism control as a driver for memory impairment upon insulin resistance. Biochem Soc Trans. 2023;51(1):287–301. doi: 10.1042/BST20220789 36606696

[7] Ehtewish H, Arredouani A, El-Agnaf O. Diagnostic, prognostic, and mechanistic biomarkers of diabetes mellitus-associated cognitive decline. Int J Mol Sci. 2022;23(11):61144. doi: 10.3390/ijms23116144 PMC9181591 35682821

[8] Secnik J, Cermakova P, Fereshtehnejad S-M, Dannberg P, Johnell K, Fastbom J, et al. Diabetes in a Large Dementia Cohort: Clinical Characteristics and Treatment From the Swedish Dementia Registry. Diabetes Care. 2017;40(9):1159–66. doi: 10.2337/dc16-2516 28655740 PMC5566285

[9] Dao L, Choi S, Freeby M. Type 2 diabetes mellitus and cognitive function: understanding the connections. Curr Opin Endocrinol Diabetes Obes. 2023;30(1):7–13. doi: 10.1097/MED.0000000000000783 36385094

[10] Schwartz SS, Herman ME, Tun MTH, Barone E, Butterfield DA. The double life of glucose metabolism: brain health, glycemic homeostasis, and your patients with type 2 diabetes. BMC Med. 2024;22(1):582. doi: 10.1186/s12916-024-03763-8 39696300 PMC11657227

[11] Neale A, Axelrod J, Geary E, Guidotti Breting LM. Mild Cognitive Impairment. Dementia. Oxford University PressNew York. 2024. p. 97–110. doi: 10.1093/med/9780197690024.003.0008

[12] Imfeld P, Bodmer M, Jick SS, Meier CR. Metformin, other antidiabetic drugs, and risk of Alzheimer’s disease: a population-based case-control study. J Am Geriatr Soc. 2012;60(5):916–21. doi: 10.1111/j.1532-5415.2012.03916.x 22458300

[13] Ha J, Choi D-W, Kim KJ, Cho SY, Kim H, Kim KY, et al. Association of metformin use with Alzheimer’s disease in patients with newly diagnosed type 2 diabetes: a population-based nested case-control study. Sci Rep. 2021;11(1):24069. doi: 10.1038/s41598-021-03406-5 34912022 PMC8674300

[14] Song J, Kim Y, Han K. Association between metformin and Alzheimer’s disease: a systematic review and meta-analysis of clinical observational studies. Front Endocrinol. 2022;13:917740. doi: 10.3389/fendo.2022.917740 35786654

[15] Tahmi M, Luchsinger JA. Metformin in the Prevention of Alzheimer’s Disease and Alzheimer’s Disease Related Dementias. J Prev Alzheimers Dis. 2023;10(4):706–17. doi: 10.14283/jpad.2023.113 37874091

[16] Doran W, Tunnicliffe L, Muzambi R, Rentsch CT, Bhaskaran K, Smeeth L, et al. Incident dementia risk among patients with type 2 diabetes receiving metformin versus alternative oral glucose-lowering therapy: an observational cohort study using UK primary healthcare records. BMJ Open Diabetes Res Care. 2024;12(1):e003548. doi: 10.1136/bmjdrc-2023-003548 38272537 PMC10823924

[17] Kuate Defo A, Bakula V, Pisaturo A, Labos C, Wing SS, Daskalopoulou SS. Diabetes, antidiabetic medications and risk of dementia: A systematic umbrella review and meta-analysis. Diabetes Obes Metab. 2024;26(2):441–62. doi: 10.1111/dom.15331 37869901

[18] Li Z, Lin C, Cai X, Lv F, Yang W, Ji L. Anti-diabetic agents and the risks of dementia in patients with type 2 diabetes: a systematic review and network meta-analysis of observational studies and randomized controlled trials. Alzheimers Res Ther. 2024;16(1):272. doi: 10.1186/s13195-024-01645-y 39716328 PMC11668108

[19] Mui JV, Zhou J, Lee S, Leung KSK, Lee TTL, Chou OHI, et al. Sodium-Glucose Cotransporter 2 (SGLT2) Inhibitors vs. Dipeptidyl Peptidase-4 (DPP4) Inhibitors for New-Onset Dementia: A Propensity Score-Matched Population-Based Study With Competing Risk Analysis. Front Cardiovasc Med. 2021;8:747620. doi: 10.3389/fcvm.2021.747620 34746262 PMC8566991

[20] Olukorode JO, Orimoloye DA, Nwachukwu NO, Onwuzo CN, Oloyede PO, Fayemi T, et al. Recent Advances and Therapeutic Benefits of Glucagon-Like Peptide-1 (GLP-1) Agonists in the Management of Type 2 Diabetes and Associated Metabolic Disorders. Cureus. 2024;16(10):e72080. doi: 10.7759/cureus.72080 39574978 PMC11579408

[21] Pelle MC, Zaffina I, Giofrè F, Pujia R, Arturi F. Potential Role of Glucagon-like Peptide-1 Receptor Agonists in the Treatment of Cognitive Decline and Dementia in Diabetes Mellitus. Int J Mol Sci. 2023;24(14):11301. doi: 10.3390/ijms241411301 37511061 PMC10379573

[22] Cui Y, Tang T-Y, Lu C-Q, Ju S. Insulin Resistance and Cognitive Impairment: Evidence From Neuroimaging. J Magn Reson Imaging. 2022;56(6):1621–49. doi: 10.1002/jmri.28358 35852470

[23] Hui EK, Mukadam N, Kohl G, Livingston G. Effect of diabetes medications on the risk of developing dementia, mild cognitive impairment, or cognitive decline: A systematic review and meta-analysis. J Alzheimers Dis. 2025;104(3):627–48. doi: 10.1177/13872877251319054 40017057 PMC12231844

[24] McMillan JM, Mele BS, Hogan DB, Leung AA. Impact of pharmacological treatment of diabetes mellitus on dementia risk: systematic review and meta-analysis. BMJ Open Diabetes Res Care. 2018;6(1):e000563. doi: 10.1136/bmjdrc-2018-000563 30487973 PMC6254737

[25] Secnik J, Xu H, Schwertner E, Hammar N, Alvarsson M, Winblad B, et al. The association of antidiabetic medications and Mini-Mental State Examination scores in patients with diabetes and dementia. Alzheimers Res Ther. 2021;13(1):197. doi: 10.1186/s13195-021-00934-0 34857046 PMC8641148

[26] Hong C-T, Chen J-H, Hu C-J. Role of glucagon-like peptide-1 receptor agonists in Alzheimer’s disease and Parkinson’s disease. J Biomed Sci. 2024;31(1):102. doi: 10.1186/s12929-024-01090-x 39501255 PMC11539687

[27] Tang H, Shao H, Shaaban CE, Yang K, Brown J, Anton S, et al. Newer glucose-lowering drugs and risk of dementia: A systematic review and meta-analysis of observational studies. J Am Geriatr Soc. 2023;71(7):2096–106. doi: 10.1111/jgs.18306 36821780 PMC10363181

[28] Samaras K, Makkar S, Crawford JD, Kochan NA, Wen W, Draper B, et al. Metformin Use Is Associated With Slowed Cognitive Decline and Reduced Incident Dementia in Older Adults With Type 2 Diabetes: The Sydney Memory and Ageing Study. Diabetes Care. 2020;43(11):2691–701. doi: 10.2337/dc20-0892 32967921

[29] Sun M, Chen W-M, Wu S-Y, Zhang J. Metformin in elderly type 2 diabetes mellitus: dose-dependent dementia risk reduction. Brain. 2024;147(4):1474–82. doi: 10.1093/brain/awad366 37878862

[30] Zhou J-B, Tang X, Han M, Yang J, Simó R. Impact of antidiabetic agents on dementia risk: A Bayesian network meta-analysis. Metabolism. 2020;109:154265. doi: 10.1016/j.metabol.2020.154265 32446679

[31] Lennox R, Porter DW, Flatt PR, Holscher C, Irwin N, Gault VA. Comparison of the independent and combined effects of sub-chronic therapy with metformin and a stable GLP-1 receptor agonist on cognitive function, hippocampal synaptic plasticity and metabolic control in high-fat fed mice. Neuropharmacology. 2014;86:22–30. doi: 10.1016/j.neuropharm.2014.06.026 24998752

[32] Youn YJ, Kim S, Jeong H-J, Ah Y-M, Yu YM. Sodium-glucose cotransporter-2 inhibitors and their potential role in dementia onset and cognitive function in patients with diabetes mellitus: a systematic review and meta-analysis. Front Neuroendocrinol. 2024;73:101131. doi: 10.1016/j.yfrne.2024.101131 38367940

[33] Tang C, Hao J, Tao F, Feng Q, Song Y, Zeng B. Association of Metformin use with risk of dementia in patients with type 2 diabetes: A systematic review and meta-analysis. Diabetes Obes Metab. 2025;27(4):1992–2001. doi: 10.1111/dom.16192 39780315

[34] Jaiswal V, Mashkoor Y, Raj N, Rajak K, Jaiswal A, Fonarow GC. Association Between SGLT2 Inhibitors and Risk of Dementia and Parkinson’s Disease: A Meta-Analysis of 12 Randomized Controlled Trials. Am J Med. 2024;137(11):1136–41. doi: 10.1016/j.amjmed.2024.06.030 38977148

[35] Pai Y-W, Chen I-C, Lin J-F, Chen X-H, Chen H-H, Chang M-H, et al. Association of sodium-glucose cotransporter 2 inhibitors with risk of incident dementia and all-cause mortality in older patients with type 2 diabetes: A retrospective cohort study using the TriNetX US collaborative networks. Diabetes Obes Metab. 2024;26(11):5420–30. doi: 10.1111/dom.15918 39248211

[36] Langan RC, Goodbred AJ. Vitamin B12 Deficiency: Recognition and Management. Am Fam Physician. 2017;96(6):384–9. 28925645

[37] Zhang Q, Li S, Li L, Li Q, Ren K, Sun X, et al. Metformin Treatment and Homocysteine: A Systematic Review and Meta-Analysis of Randomized Controlled Trials. Nutrients. 2016;8(12):798. doi: 10.3390/nu8120798 27941660 PMC5188453

[38] Mattishent K, Thavarajah M, Blanco P. Meta-analysis: association between hypoglycaemia and serious adverse events in older patients treated with glucose-lowering agents. Diabetes Obes Metab. 2016;18(10):1093–8. doi: 10.1111/dom.1258727484021

[39] Yaffe K, Falvey CM, Hamilton N, Harris TB, Simonsick EM, Strotmeyer ES, et al. Association between hypoglycemia and dementia in a biracial cohort of older adults with diabetes mellitus. JAMA Intern Med. 2013;173(14):1300–6. doi: 10.1001/jamainternmed.2013.6176 23753199 PMC4041621

[40] Mohammadi M, Moosaie F, Abdollahi A, et al. Association between severe hypoglycaemic episodes and risk of dementia in patients with type 2 diabetes mellitus: a systematic review and meta-analysis. Diabetes Metab Res Rev. 2023;39(4):e3610. doi: 10.1002/dmrr.3610 36649373

