# Supplementary material for: Antidiabetic medications and risk of cognitive disorders in type 2 diabetes: A retrospective cohort study
Source: PLoS One. 2026 Aug 24;21(8):e0356138. doi: 10.1371/journal.pone.0356138 (PMC13502699; doi:10.1371/journal.pone.0356138)
Supplement: Supplementary file 1 [file pone.0356138.s001.docx]

Table S1: Baseline characteristics: Metformin and DDP-4 inhibitors (Group 2) vs Metformin only (Group 1).

| **Groups** |  | **Before propensity score matching** | | | | **After propensity score matching** | | | |
| --- | --- | --- | --- | --- | --- | --- | --- | --- | --- |
|  | **Demographics** | **Mean** ±  **SD** | **Patients** | **P**  **Value** | **Std**  **diff.** | **Mean** ±  **SD** | **Patients** | **p-Value** | **Std**  **diff.** |
| 1 | Current Age | 58.0 +/- 7.9 | 446,109 (100) | <0.001 | 0.195 | 59.8 +/- 7.3 | 43,38 (100) | <0.001 | 0.033 |
| 2 |  | 59.5 +/- 7.4 | 43,519 (100) |  |  | 59.5 +/- 7.4 | 43,383 (100) |  |  |
| 1 | Age at Index | 52.6 +/- 8.5 | 446,109 (100) | <0.001 | 0.126 | 53.8 +/- 8.0 | 43,383 (100) | <0.001 | 0.026 |
| 2 |  | 53.6 +/- 8.0 | 43,519 (100) |  |  | 53.6 +/- 8.0 | 43,383 (100) |  |  |
| 1 | Female |  | 218,812 (49) | <0.001 | 0.064 |  | 19,663 (45.3) | 0.094 | 0.011 |
| 2 |  |  | 19,952 (45.8) |  |  |  | 19,909 (45.9) |  |  |
| 1 | Black or African American |  | 83,775 (18.8) | <0.001 | 0.036 |  | 7,395 (17) | 0.177 | 0.009 |
| 2 |  |  | 7,564 (17.4) |  |  |  | 7,545 (17.4) |  |  |
| 1 | Male |  | 219,326 (49.2) | <0.001 | 0.070 |  | 23,025 (53.1) | 0.185 | 0.009 |
| 2 |  |  | 22,923 (52.7) |  |  |  | 22,830 (52.6) |  |  |
| 1 | White |  | 243,968 (54.7) | <0.001 0.047 |  |  | 22,560 (52) | 0.305 | 0.007 |
| 2 |  |  | 22,779 (52.3) |  |  |  | 22,711 (52.3) |  |  |
| 1 | American Indian or Alaska Native |  | 2,860 (0.6) | 0.567 | 0.003 |  | 243 (0.6) | 0.050 | 0.013 |
| 2 |  |  | 289 (0.7) |  |  |  | 288 (0.7) |  |  |
| 1 | Hispanic or  Latino |  | 68,064 (15.3) | <0.001 0.027 |  |  | 5,598 (12.9) | <0.001 | 0.040 |
| 2 |  |  | 6,221 (14.3) |  |  |  | 6,194 (14.3) |  |  |
| 1 | Asian |  | 27,578 (6.2) | <0.001 0.073 |  |  | 3,684 (8.5) | 0.017 | 0.016 |
| 2 |  |  | 3,506 (8.1) |  |  |  | 3,490 (8) |  |  |
|  | | | | | | | | | |
| **Groups** | **Diagnosis** | **Mean** ±  **SD** | **Patients** | **P**  **Value** | **Std**  **diff.** | **Mean** ±  **SD** | **Patients** | **p-Value** | **Std**  **diff.** |
| 1 | Sleep apnea |  | 43,535 (9.8) | <0.001 | 0.034 |  | 3,180 (7.3) | <0.001 | 0.053 |
| 2 |  |  | 3,817 (8.8) |  |  |  | 3,801 (8.8) |  |  |
| 1 | Hypothyroidism |  | 28,288 (6.3) | 0.727 | 0.002 |  | 2,443 (5.6) | <0.001 | 0.028 |
| 2 |  |  | 2,741 (6.3) |  |  |  | 2,734 (6.3) |  |  |
| 1 | Tobacco use |  | 13,139 (2.9) | 0.005 | 0.014 |  | 1,007 (2.3) | <0.001 | 0.024 |
| 2 |  |  | 1,179 (2.7) |  |  |  | 1,170 (2.7) |  |  |
| 1 | Alcohol abuse |  | 6,834 (1.5) | <0.001 | 0.042 |  | 362 (0.8) | 0.001 | 0.023 |
| 2 |  |  | 459 (1.1) |  |  |  | 459 (1.1) |  |  |
| 1 | injuries of head |  | 7,837 (1.8) | <0.001 | 0.032 |  | 494 (1.1) | 0.003 | 0.020 |
| 2 |  |  | 593 (1.4) |  |  |  | 591 (1.4) |  |  |
|  | | | | | | | | | |
| **Groups** | **Medication** | **Mean** ±  **SD** | **Patients** | **P**  **Value** | **Std**  **diff.** | **Mean** ±  **SD** | **Patients** | **p-Value** | **Std**  **diff.** |
| 1 | Glucocorticoids |  | 128,059 (28.7) | 0.003 | 0.015 |  | 11,662 (26.9) | <0.001 | 0.026 |
| 2 |  |  | 12,203 (28) |  |  |  | 12,157 (28) |  |  |
| **Groups** |  | **Mean** ±  **SD** | **Patients** | **P**  **Value** | **Std**  **diff.** | **Mean** ±  **SD** | **Patients** | **p-Value** | **Std**  **diff.** |
| 1 | Hemoglobin  A1c | 7.1 +/- 1.73 | 190,513 (41.2) | <0.001 | 0.527 | 7.85 +/- 1.8 | 21,339 (46.6) | <0.001 | 0.164 |
| 2 |  | 8.1 +/- 1.94 | 21,345 (46.8) |  |  | 8.16 +/- 1.9 | 21,339 (46.8) |  |  |

| 1 | At most 6.50 % |  | 123,332(26.6) | <0.001 | 0.106 |  | 8,868(19.4) | <0.001 | 0.066 |
| --- | --- | --- | --- | --- | --- | --- | --- | --- | --- |
| 2 |  |  | 10,089(22.1) |  |  |  | 10,089(22.1) |  |  |
| 1 | 6.50 - 7 % |  | 79,079(17.1) | <0.001 | 0.107 |  | 8,767(19.2) | <0.001 | 0.052 |
| 2 |  |  | 9,738(21.3) |  |  |  | 9,736(21.3) |  |  |
| 1 | At least 7 % |  | 88,059(19) | <0.001 | 0.432 |  | 16,514(36.2) | <0.001 | 0.039 |
| 2 |  |  | 17,397(38.1) |  |  |  | 17,391(38.1) |  |  |
