# Supplementary material for: Antidiabetic medications and risk of cognitive disorders in type 2 diabetes: A retrospective cohort study
Source: PLoS One. 2026 Aug 24;21(8):e0356138. doi: 10.1371/journal.pone.0356138 (PMC13502699; doi:10.1371/journal.pone.0356138)
Supplement: Supplementary file 2 [file pone.0356138.s002.docx]

Table S2: Baseline characteristics: Metformin and GLP-1 receptor agonists (Group 3) vs Metformin only (Group 1).

| **Groups** |  | **Before propensity score matching** | | | | **After propensity score matching** | | | |
| --- | --- | --- | --- | --- | --- | --- | --- | --- | --- |
|  | **Demographics** | **Mean** ±  **SD** | **Patients** | **P**  **Value** | **Std**  **diff.** | **Mean** ±  **SD** | **Patients** | **p-Value** | **Std**  **diff.** |
| 1 | Current Age | 58.0 +/- 7.9 | 446,109 (100) | <0.001 | 0.251 | 56.4 +/- 8.1 | 102,399 (100) | <0.001 | 0.021 |
| 3 |  | 56.0 +/- 8.0 | 115,362 (100) |  |  | 56.2 +/- 7.9 | 102,399 (100) |  |  |
| 1 | Age at Index | 52.6 +/- 8.5 | 446,109 (100) | <0.001 | 0.085 | 53.6 +/- 8.3 | 102,399 (100) | <0.001 | 0.026 |
| 3 |  | 53.3 +/- 8.1 | 115,362 (100) |  |  | 53.4 +/- 8.1 | 102,399 (100) |  |  |
| 1 | Female |  | 218,812 (49) | <0.001 | 0.217 |  | 59,674 (58.3) | 0.167 | 0.006 |
| 3 |  |  | 68,964 (59.8) |  |  |  | 59,982 (58.6) |  |  |
| 1 | Black or African American |  | 83,775 (18.8) | <0.001 | 0.030 |  | 17,577 (17.2) | <0.001 | 0.019 |
| 3 |  |  | 20,337 (17.6) |  |  |  | 18,333 (17.9) |  |  |
| 1 | Male |  | 219,326 (49.2) | <0.001 | 0.242 |  | 39,639 (38.7) | 0.462 | 0.003 |
| 3 |  |  | 42,972 (37.2) |  |  |  | 39,477 (38.6) |  |  |
| 1 | White |  | 243,968 (54.7) | <0.001 | 0.161 |  | 63,951 (62.5) | <0.001 | 0.023 |
| 3 |  |  | 72,229 (62.6) |  |  |  | 62,828 (61.4) |  |  |
| 1 | American Indian or Alaska Native |  | 2,860 (0.6) | 0.001 | 0.011 |  | 540 (0.5) | 0.198 | 0.006 |
| 3 |  |  | 640 (0.6) |  |  |  | 583 (0.6) |  |  |
| 1 | Hispanic or  Latino |  | 68,064 (15.3) | <0.001 | 0.173 |  | 9,314 (9.1) | <0.001 | 0.038 |
| 3 |  |  | 11,059 (9.6) |  |  |  | 10,449 (10.2) |  |  |
| 1 | Asian |  | 27,578 (6.2) | <0.001 | 0.113 |  | 3,916 (3.8) | 0.006 | 0.012 |
| 3 |  |  | 4,309 (3.7) |  |  |  | 4,159 (4.1) |  |  |
|  | | | | | | | | | |
| **Groups** | **Diagnosis** | **Mean** ±  **SD** | **Patients** | **P**  **Value** | **Std**  **diff.** | **Mean** ±  **SD** | **Patients** | **p-Value** | **Std**  **diff.** |
| 1 | Sleep apnea |  | 43,535 (9.8) | <0.001 | 0.444 |  | 22,566 (22) | 0.002 | 0.014 |
| 3 |  |  | 30,522 (26.5) |  |  |  | 23,144 (22.6) |  |  |
| 1 | Hypothyroidism |  | 28,288 (6.3) | <0.001 | 0.231 |  | 12,158 (11.9) | 0.618 | 0.002 |
| 3 |  |  | 15,151 (13.1) |  |  |  | 12,085 (11.8) |  |  |
| 1 | Tobacco use |  | 13,139 (2.9) | <0.001 | 0.099 |  | 4,380 (4.3) | <0.001 | 0.018 |
| 3 |  |  | 5,600 (4.9) |  |  |  | 4,769 (4.7) |  |  |
| 1 | Alcohol abuse |  | 6,834 (1.5) | 0.003 | 0.010 |  | 1,283 (1.3) | <0.001 | 0.017 |
| 3 |  |  | 1,628 (1.4) |  |  |  | 1,487 (1.5) |  |  |
| 1 | injuries of head |  | 7,837 (1.8) | <0.001 | 0.059 |  | 2,311 (2.3) | 0.001 | 0.015 |
| 3 |  |  | 3,017 (2.6) |  |  |  | 2,546 (2.5) |  |  |
|  | | | | | | | | | |
| **Groups** | **Medication** | **Mean** ±  **SD** | **Patients** | **P**  **Value** | **Std**  **diff.** | **Mean** ±  **SD** | **Patients** | **p-Value** | **Std**  **diff.** |
| 1 | Glucocorticoids |  | 128,059 (28.7) | <0.001 | 0.528 |  | 52,272 (51) | 0.024 | 0.010 |
| 3 |  |  | 62,096 (53.8) |  |  |  | 51,761 (50.5) |  |  |
| **Groups** |  | **Mean** ±  **SD** | **Patients** | **P**  **Value** | **Std**  **diff.** | **Mean** ±  **SD** | **Patients** | **p-Value** | **Std**  **diff.** |
| 1 | Hemoglobin  A1c | 7.19 +/- 1.75 | 190,513 (41.2) | <0.001 | 0.0651 | 7.29 +/- 1.78 | 71,345 (67.5) | 0.0001 | 0.0211 |
| 3 |  | 7.31 +/- 1.75 | 86,421 (72) |  |  | 7.26 +/- 1.68 | 72,363 (68.5) |  |  |
| 1 | At most 6.50 % |  | 123,332(26.6) | <0.001 | 0.545 |  | 50,952 (48.8) | 0.815 | 0.010 |
| 3 |  |  | 62,843(52.4) |  |  |  | 50,952 (48.1) |  |  |

| 1 | 6.5-7 % |  | 79,079(17.7) | <0.001 | 0.456 |  | 32,570(32.3) | 0.0001 | 0.0320 |
| --- | --- | --- | --- | --- | --- | --- | --- | --- | --- |
| 3 |  |  | 43,991(38.1) |  |  |  | 34,141(30.8) |  |  |
| 1 | At least 7 % |  | 88,059(19) | <0.001 | 0.5134 |  | 38,308(36.2) | 0.0003 | 0.0157 |
| 3 |  |  | 50,302(41.9) |  |  |  | 39,107(37) |  |  |
