# Supplementary material for: Antidiabetic medications and risk of cognitive disorders in type 2 diabetes: A retrospective cohort study
Source: PLoS One. 2026 Aug 24;21(8):e0356138. doi: 10.1371/journal.pone.0356138 (PMC13502699; doi:10.1371/journal.pone.0356138)
Supplement: Supplementary file 3 [file pone.0356138.s003.docx]

Table S3: Baseline characteristics: Metformin and SGLT-2 inhibitors (Group 4) vs Metformin only (Group 1).

| **Groups** |  | **Before propensity score matching** | | | | **After propensity score matching** | | | |
| --- | --- | --- | --- | --- | --- | --- | --- | --- | --- |
|  | **Demographics** | **Mean** ±  **SD** | **Patients** | **P**  **Value** | **Std**  **diff.** | **Mean** ±  **SD** | **Patients** | **p-Value** | **Std**  **diff.** |
| 1 | Current Age | 58.0 +/- 7.9 | 446,109 (100) | <0.001 | 0.072 | 58.7 +/- 7.6 | 58,327 (100) | 0.002 | 0.018 |
| 4 |  | 58.6 +/- 7.5 | 59,691 (100) |  |  | 58.6 +/- 7.5 | 58,327 (100) |  |  |
| 1 | Age at Index | 52.6 +/- 8.5 | 446,109 (100) | <0.001 | 0.382 | 55.9 +/- 7.8 | 58,327 (100) | <0.001 | 0.030 |
| 4 |  | 55.7 +/- 7.8 | 59,691 (100) |  |  | 55.6 +/- 7.8 | 58,327 (100) |  |  |
| 1 | Female |  | 218,812 (49) | <0.001 | 0.239 |  | 21,468 (36.8) | 0.001 | 0.019 |
| 4 |  |  | 22,249 (37.3) |  |  |  | 22,004 (37.7) |  |  |
| 1 | Black or African American |  | 83,775 (18.8) | <0.001 | 0.102 |  | 8,231 (14.1) | <0.001 | 0.026 |
| 4 |  |  | 8,922 (14.9) |  |  |  | 8,760 (15) |  |  |
| 1 | Male |  | 219,326 (49.2) | <0.001 | 0.222 |  | 35,235 (60.4) | 0.026 | 0.013 |
| 4 |  |  | 35,916 (60.2) |  |  |  | 34,863 (59.8) |  |  |
| 1 | White |  | 243,968 (54.7) | <0.001 0.032 |  |  | 33,737 (57.8) | <0.001 | 0.031 |
| 4 |  |  | 33,596 (56.3) |  |  |  | 32,831 (56.3) |  |  |
| 1 | American Indian or Alaska Native |  | 2,860 (0.6) | <0.001 0.020 |  |  | 244 (0.4) | 0.038 | 0.012 |
| 4 |  |  | 295 (0.5) |  |  |  | 292 (0.5) |  |  |
| 1 | Hispanic or  Latino |  | 68,064 (15.3) | <0.001 0.080 |  |  | 6,669 (11.4) | <0.001 | 0.032 |
| 4 |  |  | 7,456 (12.5) |  |  |  | 7,277 (12.5) |  |  |
| 1 | Asian |  | 27,578 (6.2) | <0.001 0.097 |  |  | 4,847 (8.3) | 0.030 | 0.013 |
| 4 |  |  | 5,206 (8.7) |  |  |  | 5,053 (8.7) |  |  |
|  | | | | | | | | | |
| **Groups** | **Diagnosis** | **Mean** ±  **SD** | **Patients** | **P**  **Value** | **Std**  **diff.** | **Mean** ±  **SD** | **Patients** | **p-Value** | **Std**  **diff.** |
| 1 | Sleep apnea |  | 43,535 (9.8) | <0.001 | 0.150 |  | 8,003 (13.7) | <0.001 | 0.023 |
| 4 |  |  | 8,747 (14.7) |  |  |  | 8,474 (14.5) |  |  |
| 1 | Hypothyroidism |  | 28,288 (6.3) | <0.001 | 0.038 |  | 3,883 (6.7) | <0.001 | 0.025 |
| 4 |  |  | 4,362 (7.3) |  |  |  | 4,248 (7.3) |  |  |
| 1 | Tobacco use |  | 13,139 (2.9) | <0.001 | 0.075 |  | 2,253 (3.9) | <0.001 | 0.021 |
| 4 |  |  | 2,592 (4.3) |  |  |  | 2,493 (4.3) |  |  |
| 1 | Alcohol abuse |  | 6,834 (1.5) | 0.324 | 0.004 |  | 769 (1.3) | <0.001 | 0.022 |
| 4 |  |  | 946 (1.6) |  |  |  | 919 (1.6) |  |  |
| 1 | injuries of head |  | 7,837 (1.8) | 0.167 | 0.006 |  | 986 (1.7) | 0.056 | 0.011 |
| 4 |  |  | 1,096 (1.8) |  |  |  | 1,072 (1.8) |  |  |
|  | | | | | | | | | |
| **Groups** | **Medication** | **Mean** ±  **SD** | **Patients** | **P**  **Value** | **Std**  **diff.** | **Mean** ±  **SD** | **Patients** | **p-Value** | **Std**  **diff.** |
| 1 | Glucocorticoids |  | 128,059 (28.7) | <0.001 | 0.191 |  | 21,905 (37.6) | 0.561 | 0.003 |
| 4 |  |  | 22,466 (37.6) |  |  |  | 21,809 (37.4) |  |  |
| **Groups** |  | **Mean** ±  **SD** | **Patients** | **P**  **Value** | **Std**  **diff.** | **Mean** ±  **SD** | **Patients** | **p-Value** | **Std**  **diff.** |
| 1 | Hemoglobin  A1c | 7.19 +/- 1.7 | 190,513 (41.2) | <0.001 | 0.471 | 7.7 +/- 1.7 | 34,342 (56.6) | <0.001 | 0.152 |
| 4 |  | 8.0 +/- 1.8 | 35,037 (57.1) |  |  | 8.0 +/- 1.8 | 34,342 (56.6) |  |  |
| 1 | At most 6.50 % |  | 123,332 (26.6) | <0.001 | 0.080 |  | 17,262 (26.5) | <0.001 | 0.039 |
| 4 |  |  | 18,585 (30.3) |  |  |  | 18,348 (28.7) |  |  |

| 1 | 6.50 - 7 % |  | 79,079(17.1) | <0.001 | 0.288 |  | 16,092(26.5) | <0.001 | 0.049 |
| --- | --- | --- | --- | --- | --- | --- | --- | --- | --- |
| 4 |  |  | 17,877(29.1) |  |  |  | 17,426(28.7) |  |  |
| 1 | At least 7 % |  | 88,05(19) | <0.001 | 0.590 |  | 25,835(42.6) | <0.001 | 0.046 |
| 4 |  |  | 27,91(45.5) |  |  |  | 27,226(44.9) |  |  |
