# Supplementary material for: Antidiabetic medications and risk of cognitive disorders in type 2 diabetes: A retrospective cohort study
Source: PLoS One. 2026 Aug 24;21(8):e0356138. doi: 10.1371/journal.pone.0356138 (PMC13502699; doi:10.1371/journal.pone.0356138)
Supplement: Supplementary file 4 [file pone.0356138.s004.docx]

Table S4: Baseline characteristics: Insulin only (Group 5) vs Metformin only (Group 1):

| **Groups** |  | **Before propensity score matching** | | | | **After propensity score matching** | | | |
| --- | --- | --- | --- | --- | --- | --- | --- | --- | --- |
|  | **Demographics** | **Mean** ±  **SD** | **Patients** | **P-Value** | **Std**  **diff.** | **Mean** ±  **SD** | **Patients** | **p-Value** | **Std**  **diff.** |
| 1 | Current Age | 58.0 +/- 7.9 | 446,109 (100) | <0.001 | 0.058 | 58.3 +/- 7.9 | 333,153 (100) | 0.005 | 0.007 |
| 5 |  | 58.5 +/- 7.9 | 368,668 (100) |  |  | 58.3 +/- 8.0 | 333,153 (100) |  |  |
| 1 | Age at Index | 52.6 +/- 8.5 | 446,109 (100) | <0.001 | 0.089 | 52.9 +/- 8.4 | 333,153 (100) | <0.001 | 0.011 |
| 5 |  | 53.3 +/- 8.6 | 368,668 (100) |  |  | 53.0 +/- 8.6 | 333,153 (100) |  |  |
| 1 | Female |  | 218,812 (49) | <0.001 | 0.054 |  | 158,871 (47.7) | 0.026 | 0.005 |
| 5 |  |  | 170,802 (46.3) |  |  |  | 157,963 (47.4) |  |  |
| 1 | Black or African American |  | 83,775 (18.8) | <0.001 | 0.019 |  | 65,894 (19.8) | 0.447 | 0.002 |
| 5 |  |  | 71,917 (19.5) |  |  |  | 65,647 (19.7) |  |  |
| 1 | Male |  | 219,326 (49.2) | <0.001 | 0.039 |  | 167,786 (50.4) | 0.619 | 0.001 |
| 5 |  |  | 188,412 (51.1) |  |  |  | 167,989 (50.4) |  |  |
| 1 | White |  | 243,968 (54.7) | <0.001 | 0.087 |  | 191,820 (57.6) | 0.129 | 0.004 |
| 5 |  |  | 217,501 (59) |  |  |  | 192,433 (57.8) |  |  |
| 1 | American Indian or Alaska Native |  | 2,860 (0.6) | <0.001 | 0.033 |  | 2,489 (0.7) | 0.011 | 0.006 |
| 5 |  |  | 3,450 (0.9) |  |  |  | 2,670 (0.8) |  |  |
| 1 | Hispanic or  Latino |  | 68,064 (15.3) | <0.001 | 0.090 |  | 43,687 (13.1) | 0.387 | 0.002 |
| 5 |  |  | 44,896 (12.2) |  |  |  | 43,449 (13) |  |  |
| 1 | Asian |  | 27,578 (6.2) | <0.001 | 0.099 |  | 14,477 (4.3) | 0.635 | 0.001 |
| 5 |  |  | 14,765 (4.0) |  |  |  | 14,556 (4.4) |  |  |
|  | | | | | | | | | |
| **Groups** | **Diagnosis** | **Mean** ±  **SD** | **Patients** | **P-Value** | **Std**  **diff.** | **Mean** ±  **SD** | **Patients** | **p-Value** | **Std**  **diff.** |
| 1 | Sleep apnea |  | 43,535 (9.8) | 0.004 | 0.006 |  | 30,299 (9.1) | <0.001 | 0.013 |
| 5 |  |  | 36,677 (9.9) |  |  |  | 31,531 (9.5) |  |  |
| 1 | Hypothyroidism |  | 28,288 (6.3) | <0.001 | 0.009 |  | 18,489 (5.5) | <0.001 | 0.014 |
| 5 |  |  | 22,595 (6.1) |  |  |  | 19,584 (5.9) |  |  |
| 1 | Tobacco use |  | 13,139 (2.9) | <0.001 | 0.048 |  | 9,909 (3.0) | <0.001 | 0.011 |
| 5 |  |  | 14,063 (3.8) |  |  |  | 10,534 (3.2) |  |  |
| 1 | Alcohol abuse |  | 6,834 (1.5) | <0.001 | 0.117 |  | 6,302 (1.9) | <0.001 | 0.014 |
| 5 |  |  | 12,262 (3.3) |  |  |  | 6,972 (2.1) |  |  |
| 1 | injuries of head |  | 7,837 (1.8) | <0.001 | 0.058 |  | 6,464 (1.9) | <0.001 | 0.011 |
| 5 |  |  | 9,611 (2.6) |  |  |  | 6,997 (2.1) |  |  |
|  | | | | | | | | | |
| **Groups** | **Medication** | **Mean** ±  **SD** | **Patients** | **P-Value** | **Std**  **diff.** | **Mean** ±  **SD** | **Patients** | **p-Value** | **Std**  **diff.** |
| 1 | Glucocorticoids |  | 128,059 (28.7) | <0.001 | 0.053 |  | 94,836 (28.5) | <0.001 | 0.013 |
| 5 |  |  | 114,855 (31.2) |  |  |  | 96,854 (29.1) |  |  |
| **Groups** |  | **Mean** ±  **SD** | **Patients** | **P-Value** | **Std**  **diff.** | **Mean** ±  **SD** | **Patients** | **p-Value** | **Std**  **diff.** |
| 1 | Hemoglobin  A1c | 7.1 +/- 1.7 | 190,513 (41.2) | 0.009 | 0.012 | 7.2 +/- 1.8 | 101,925 (29.4) | 0.075 | 0.079 |
| 5 |  | 7.2 +/- 2.2 | 116,206 (29.8) |  |  | 7.2 +/- 2.2 | 108,563 (31.3) |  |  |
| 1 | At most 6.50 % |  | 123,332 (26.6) | <0.001 | 0.221 |  | 60,237 (17.4) | <0.001 | 0.021 |
| 5 |  |  | 68,374 (17.5) |  |  |  | 63,072 (18.2) |  |  |
| 1 | 6.50 - 7 % |  | 79,079 (17.1) | <0.001 | 0.322 |  | 25,104 (7.2) | <0.001 | 0.012 |

| 5 |  |  | 26,442(6.7) |  |  |  | 26,284(7.5) |  |  |
| --- | --- | --- | --- | --- | --- | --- | --- | --- | --- |
| 1 | At least 7 % |  | 88,059(19) | <0.001 | 0.168 |  | 47,098(13.6) | 0.006 | 0.006 |
| 5 |  |  | 50,264(12.9) |  |  |  | 47,874(13.8) |  |  |
