# Supplementary material for: Antidiabetic medications and risk of cognitive disorders in type 2 diabetes: A retrospective cohort study
Source: PLoS One. 2026 Aug 24;21(8):e0356138. doi: 10.1371/journal.pone.0356138 (PMC13502699; doi:10.1371/journal.pone.0356138)
Supplement: Supplementary file 5 [file pone.0356138.s005.docx]

**Table S5.** Cognitive outcomes in patients receiving metformin plus SGLT-2 inhibitors or insulin monotherapy compared with Metformin only, before propensity score matching.

Unmatched cohorts. Comparator group: Metformin only (N = 446,109 for all comparisons). Landmark analysis at <5 years and ≥5 years follow-up.

|  | | Metformin + DPP-4i (N = N = 43,519 unmatched) | | | | | Metformin + GLP-1 RA (N = N = 115,362 unmatched) | | | | |
| --- | --- | --- | --- | --- | --- | --- | --- | --- | --- | --- | --- |
| Period | Outcome | Cases  (DPP-4i + Metf) | Cases (Metformin) | HR | 95% CI | p | Cases  (Metf + GLP-1 RA) | Cases  (Metformin) | HR | 95% CI | p |
| <5 years | Vascular dementia | 105 | 1200 | 0.86 | (0.704–1.050) | 0.138 | 134 | 1200 | 0.46 | (0.385–0.551) | <0.001 |
|  | Mild cognitive disorder | 104 | 998 | 1.02 | (0.835–1.250) | 0.835 | 243 | 998 | 1.05 | (0.911–1.208) | 0.509 |
|  | Alzheimer disease | 24 | 318 | 0.74 | (0.490–1.123) | 0.156 | 35 | 318 | 0.46 | (0.325–0.655) | <0.001 |
| ≥5 years | Vascular dementia | 86 | 1422 | 0.84 | (0.699–1.015) | 0.071 | 145 | 1422 | 0.48 | (0.403–0.569) | <0.001 |
|  | Mild cognitive disorder | 88 | 1218 | 0.98 | (0.815–1.186) | 0.861 | 258 | 1218 | 1.06 | (0.920–1.209) | 0.443 |
|  | Alzheimer disease | 28 | 382 | 0.73 | (0.500–1.078) | 0.113 | 42 | 382 | 0.53 | (0.387–0.736) | <0.001 |

HR = hazard ratio; CI = confidence interval; DPP-4i = dipeptidyl peptidase-4 inhibitors; GLP-1 RA = glucagon-like peptide-1 receptor agonists; metf. = Metformin only. Cox proportional hazards models. Groups were not matched.
