# Supplementary material for: Antidiabetic medications and risk of cognitive disorders in type 2 diabetes: A retrospective cohort study
Source: PLoS One. 2026 Aug 24;21(8):e0356138. doi: 10.1371/journal.pone.0356138 (PMC13502699; doi:10.1371/journal.pone.0356138)
Supplement: Supplementary file 6 [file pone.0356138.s006.docx]

**Table S6.** Cognitive outcomes in patients receiving metformin plus SGLT-2 inhibitors or insulin monotherapy compared with metformin only, before propensity score matching

Unmatched cohorts. Comparator group: metformin only (N = 446,109 for all comparisons). Landmark analysis at <5 years and ≥5 years follow-up.

|  | | Metformin + SGLT-2i (N = N = 59,691 unmatched) | | | | | Insulin monotherapy (N = N = 368,668 unmatched) | | | | |
| --- | --- | --- | --- | --- | --- | --- | --- | --- | --- | --- | --- |
| Period | Outcome | Cases  (Metf + SGLT-2i) | Cases  (Metformin) | HR | 95% CI | p | Cases  (Insulin) | Cases  (Metformin) | HR | 95% CI | p |
| <5 years | Vascular dementia | 116 | 1200 | 0.84 | (0.694–1.016) | 0.073 | 3,061 | 1200 | 3.86 | (3.606–4.122) | <0.001 |
|  | Mild cognitive disorder | 134 | 998 | 1.21 | (1.010–1.451) | 0.038 | 1,238 | 998 | 1.93 | (1.774–2.097) | <0.001 |
|  | Alzheimer disease | 40 | 318 | 1.11 | (0.796–1.539) | 0.547 | 386 | 318 | 1.85 | (1.598–2.151) | <0.001 |
| ≥5 years | Vascular dementia | 119 | 1422 | 0.83 | (0.688–1.003) | 0.053 | 3,061 | 1422 | 3.86 | (3.606–4.122) | <0.001 |
|  | Mild cognitive disorder | 137 | 1218 | 1.18 | (0.988–1.411) | 0.068 | 1,238 | 1218 | 1.93 | (1.774–2.097) | <0.001 |
|  | Alzheimer disease | 41 | 382 | 1.09 | (0.789–1.511) | 0.597 | 386 | 382 | 1.85 | (1.598–2.151) | <0.001 |

HR = hazard ratio; CI = confidence interval; SGLT-2i = sodium-glucose cotransporter-2 inhibitors; Metf. = metformin. Cox proportional hazards models. Groups were not matched;
